# Transient Glyco-Engineering to Produce Recombinant IgA1 with Defined *N*- and *O*-Glycans in Plants

**DOI:** 10.3389/fpls.2016.00018

**Published:** 2016-01-29

**Authors:** Martina Dicker, Marc Tschofen, Daniel Maresch, Julia König, Paloma Juarez, Diego Orzaez, Friedrich Altmann, Herta Steinkellner, Richard Strasser

**Affiliations:** ^1^Department of Applied Genetics and Cell Biology, University of Natural Resources and Life SciencesVienna, Austria; ^2^Department of Chemistry, University of Natural Resources and Life SciencesVienna, Austria; ^3^Institute of Molecular and Cellular Plant Biology, Spanish Research Council Agency – Polytechnic University of ValenciaValencia, Spain

**Keywords:** monomeric IgA, antibody, protein glycosylation, *N*-glycosylation, *O*-glycosylation, glyco-engineering, recombinant glycoprotein, plant-made pharmaceuticals

## Abstract

The production of therapeutic antibodies to combat pathogens and treat diseases, such as cancer is of great interest for the biotechnology industry. The recent development of plant-based expression systems has demonstrated that plants are well-suited for the production of recombinant monoclonal antibodies with defined glycosylation. Compared to immunoglobulin G (IgG), less effort has been undertaken to express immunoglobulin A (IgA), which is the most prevalent antibody class at mucosal sites and a promising candidate for novel recombinant biopharmaceuticals with enhanced anti-tumor activity. Here, we transiently expressed recombinant human IgA1 against the VP8* rotavirus antigen in glyco-engineered ΔXT/FT *Nicotiana benthamiana* plants. Mass spectrometric analysis of IgA1 glycopeptides revealed the presence of complex biantennary *N*-glycans with terminal *N*-acetylglucosamine present on the *N*-glycosylation site of the CH2 domain in the IgA1 alpha chain. Analysis of the peptide carrying nine potential *O*-glycosylation sites in the IgA1 alpha chain hinge region showed the presence of plant-specific modifications including hydroxyproline formation and the attachment of pentoses. By co-expression of enzymes required for initiation and elongation of human *O*-glycosylation it was possible to generate disialylated mucin-type core 1 *O*-glycans on plant-produced IgA1. Our data demonstrate that ΔXT/FT *N. benthamiana* plants can be engineered toward the production of recombinant IgA1 with defined human-type *N*- and *O*-linked glycans.

## Introduction

Therapeutic antibodies are an increasingly important class of recombinant biopharmaceuticals for treatment of cancer or infectious diseases. Currently the majority of antibody-based therapeutics on the market or in clinical trials are monoclonal and of the IgG subtype. Immunoglobulin A (IgA) – the most prevalent antibody class at mucosal sites in the human body – is gaining attention as therapeutic agent for treatment of infections and cancer (Bakema and van Egmond, 2011; Boross et al., 2013; Reinhart and Kunert, 2015). However, the full potential of recombinant IgAs as therapeutic antibodies is still not fully explored, owing to the fact that robust recombinant production is challenging and that IgAs are extensively glycosylated proteins. IgGs contain a single *N*-glycosylation site in the heavy chain Fc region, which is heterogeneously glycosylated. Importantly, distinct IgG *N*-glycan structures can affect the antibody activity in therapeutic settings through the modulation of Fc receptor binding on different immune cells (Jefferis, 2009; Ferrara et al., 2011; Lin et al., 2015). Recent advances in glyco-engineering in diverse expression hosts allow the recombinant generation of IgG glycoforms for structure-function studies and comparison of efficacy (Strasser et al., 2014). The first glyco-engineered IgG-based monoclonal antibodies have already been approved for therapeutic applications (Beck and Reichert, 2012; Ratner, 2014; Goede et al., 2015).

**FIGURE 1:**
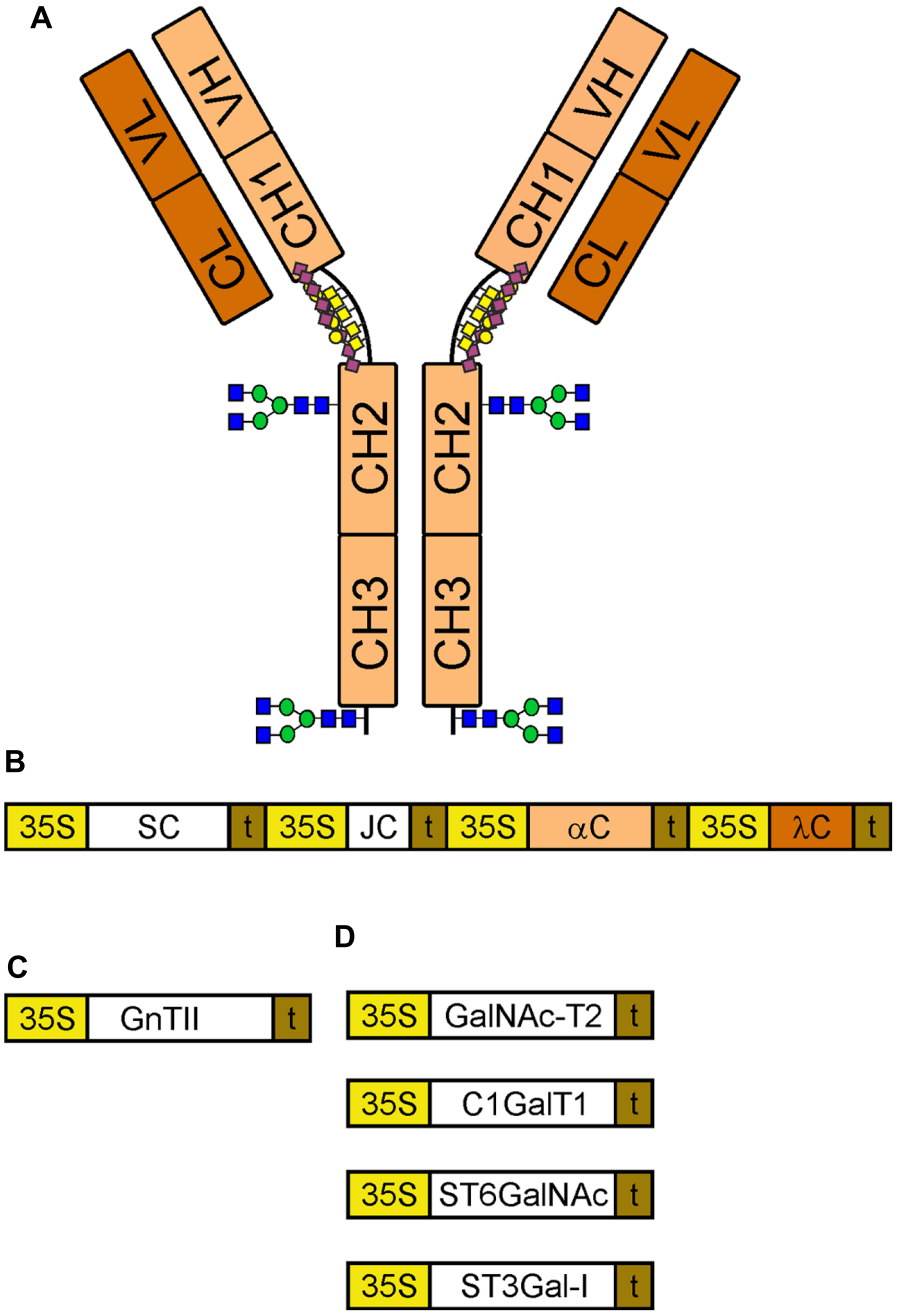
**Schematic overview of constructs for expression and glyco-engineering.** **(A)** A monomeric IgA1 with its different domains is indicated. The *N*-glycosylation sites in the CH2 domain and in the tailpiece of the alpha chain are marked using the symbols according to the nomenclature from the Consortium for Functional Glycomics (http://www.functionalglycomics.org/). The *O*-glycans in the hinge region are depicted by yellow squares (GalNAc), yellow cycles (galactose) and purple diamonds (sialic acid). **(B)** Illustration of the multigene expression vector for sIgA1. 35S, cauliflower mosaic virus 35S promoter; SC, secretory component; t, terminator sequence; JC, joining chain; αC, alpha chain; λC, lambda light chain. **(C)** GnTII: human *N*-acetylglucosaminyltransferase II used for *N*-glycan engineering. **(D)** Enzymes for sialylated core 1 formation: GalNAc-T2, human polypeptide GalNAc-transferase 2; C1GalT1, *Drosophila melanogaster* core 1 β1,3-galactosyltransferase; ST6GalNAc, *Mus musculus* α2,6-sialyltransferase III/IV; ST3Gal-I, human α2,3-sialyltransferase I.

Despite the recognized importance of *N*-glycosylation for IgG function, comparatively little is known about the role of glycosylation for the biophysical and immunological properties of IgA as well as on the *in vivo* role of different IgA glycoforms. The two IgA isotypes (IgA1 and IgA2) carry two to five *N*-glycosylation sites on the alpha chain, and the hinge region of IgA1 is modified with several mucin-type *O*-glycans (Royle et al., 2003; Deshpande et al., 2010; Huang et al., 2015) (**Figure 1A**). Aberrantly *O*-glycosylated IgA1 is involved in pathogenesis of IgA nephropathy in humans (Novak et al., 2012). In this autoimmune disease the galactose-deficient *O*-glycans in the IgA1 hinge region are recognized by circulating autoantibodies resulting in the formation of immune complexes, followed by aggregation or disposition, which is a major cause of renal failure. Moreover, the joining (J) chain in the dimeric IgA variant contains a single *N*-glycan and the secretory component (SC) in the secretory IgA (sIgA) variant is heavily *N*-glycosylated (Huang et al., 2015). Hence, the generation of recombinant IgA variants bearing homogeneous and well-defined glycans is highly challenging. Nonetheless, such glyco-engineering approaches are imperative to study the contribution of *N*- and *O*-glycosylation to IgA function. Furthermore, in the case of therapeutic applications abnormal glycosylation such as galactose-deficient IgA1 *O*-glycans variants should be avoided to reduce the risk of adverse side effects like the formation of anti-glycan antibodies (Suzuki et al., 2015).

Plants are considered attractive hosts for the production of recombinant biopharmaceuticals. For example, a phase I clinical trial of tobacco-derived HIV neutralizing antibody 2G12 has recently been completed (Ma et al., 2015). The tobacco-related species *Nicotiana benthamiana* has emerged as promising host for expression of recombinant glycoproteins with tailor-made *N*- and *O*-glycan modifications (Strasser et al., 2014). IgG variants with different types of customized *N*-glycans have been successfully generated in this expression platform (Strasser et al., 2008, 2009) and the ZMAPP^TM^ monoclonal IgG antibody cocktail for treatment of Ebola infections is produced in glyco-engineered ΔXT/FT *N. benthamiana* (Castilho et al., 2011; Qiu et al., 2014). In the ΔXT/FT *N. benthamiana* the expression of the β1,2-xylosyltransferase (XT) and core α1,3-fucosyltransferase (FT) have been downregulated by an RNAi approach (Strasser et al., 2008). In addition, mucin-type *O*-glycosylation has been generated on *N. benthamiana*-produced mucin-derived peptides and on recombinant erythropoietin fused to Fc (Castilho et al., 2012; Yang et al., 2012).

Stable expression of a recombinant IgA (CaroRX^TM^) to prevent dental caries has initially been performed in *Nicotiana tabacum* plants (Ma et al., 1995). This pioneering work demonstrated the potential of plants for the production of functional recombinant sIgA. More recently, the production of IgA variants in different plant species has been reported, but there are only few data available on the glycosylation of recombinant plant-produced IgA variants (Karnoup et al., 2005; Paul et al., 2014; Westerhof et al., 2014). Moreover, a comparison of *N*- and *O*-glycans and attempts to modulate them *in vivo* have not been described yet. In this study, we investigated the capability of glyco-engineered wild-type and ΔXT/FT *N. benthamiana* to produce recombinant IgA1 with specific glycans. We transiently expressed recombinant IgA1 against rotavirus (Juárez et al., 2012; Juarez et al., 2013) and performed an analysis of the *N*-glycan composition found in the CH2 domain as well as of the *O*-glycan structures in the hinge region of the alpha chain. Our data provide important insights for future strategies aiming at the production of IgA1 variants with customized glycosylation and enhanced therapeutic effectiveness.

## Materials and Methods

### Cloning and Expression Vectors

The human *N*-acetylglucosaminyltransferase II (GnTII) coding sequence was amplified by PCR from human cDNA (Mucha et al., 2002) with the primers Hs-GnTII1F (5′-TATATCTAGAATGAGGTTCCGCATCTACAAACG-3′) and Hs-GnTII2R (5′-tataGGATCCTCACTGCAGTCTTCTATAACT TT-3′). The PCR product was digested with XbaI/BamHI and ligated into XbaI/BamHI digested vector pPT2M (Strasser et al., 2005) to generate pPT2M-GnTII. Binary expression vectors for mucin-type *O*-glycan formation and CMP-sialic biosynthesis and Golgi transport were available from previous studies (Castilho et al., 2010, 2012). The multigene expression vector encoding the alpha chain (αC), the lambda light chain (λC), the human SC and the human joining chain (JC) was described in detail recently (Juarez et al., 2013).

### Plant Material and Transient Protein Expression

*Nicotiana benthamiana* ΔXT/FT plants which have strongly reduced expression of β1,2-XT and core α1,3-FT (Strasser et al., 2008) were grown in a growth chamber at 24°C with a 16 h light/8 h dark photoperiod. Five-week-old plants were used for syringe-mediated agroinfiltration into leaves as described previously (Strasser et al., 2008). The recombinant sIgA1 was either expressed alone or co-infiltrated with the vectors encoding the proteins for *N*-glycan modification or mucin-type *O*-glycosylation (OD_600_ of 0.2 for all agrobacteria containing sIgA1 vectors and OD_600_ of 0.05 for all constructs involved in glycan modifications).

### Protein Extraction and Purification

To purify sIgA1, 40–50 g of leaf material was harvested four days post infiltration, frozen in liquid nitrogen and disrupted using a mixer mill. The homogenized leaf material was dissolved in 2 ml extraction buffer (0.1 M Tris, 0.5 M NaCl, 1 mM EDTA, 40 mM ascorbic acid, pH 6.8) per g of plant material and incubated for 30 min at 4°C. The extract was centrifuged at 27000 × *g* for 30 min at 4°C, passed through a filter with a pore size of 12–8 μm and centrifuged again. To clear the extract it was ran through filters with pore sizes of 12–8 μm, 3–2 μm, 0.45 μm, and 0.22 μm. A chromatography column was packed with 1 ml of SSL7/Agarose (InvivoGen) and washed with 5 ml of PBS. The cleared extract was applied to the column with a flow rate of ∼1 ml/min. Afterwards the column was washed again with 5 ml PBS and the protein was eluted with 5 ml of 0.1 M glycine pH 2.5. The collected eluate fractions were immediately neutralized to pH 7.0 with 1 M Tris pH 8.0 and the protein content was analyzed using the Micro BCA Protein Assay Kit (Thermo Scientific Pierce) and bovine serum albumin (BSA) as a standard.

To isolate intercellular fluid (IF) infiltrated leaves were carefully detached and submerged in a beaker filled with buffer (0.1 M Tris pH 7.5, 10 mM MgCl_2_, 2 mM EDTA). The beaker was positioned in a desiccator and vacuum was applied for 2 min. The vacuum infiltrated leaves were inserted into a 50 ml falcon tube with a fine plain-weave cotton fabric (muslin bandage) inside to prevent damage of the leaves and centrifuged at 1000 × *g* for 20 min at 4°C. The IF was collected from the bottom of the tube and directly used for further analysis or concentrated using micro spin-columns.

### Immunoblot Analysis and Endoglycosidase Treatment

SDS-PAGE was performed in 8–10% polyacrylamide gels run under reducing or non-reducing conditions. Separated proteins were either detected by Coomassie Brilliant Blue staining or by transfer onto nitrocellulose membranes (Hybond-C, GE Healthcare) and subsequent detection with different antibodies and chemiluminescence-based detection reagents. Detection of the αC was done using a polyclonal goat anti-human alpha chain specific antibody (Sigma–Aldrich), the λC was detected using a rabbit anti-human lambda light chain antibody (Sigma–Aldrich) and the SC was detected using a rabbit anti-human SC antibody (Gentaur).

Crude protein extracts, SSL7-prufied sIgA1 or IF fractions were subjected to enzymatic deglycosylation. For endoglycosidase H (Endo H) digestion 1.5 μl of 10x Glycoprotein Denaturing Buffer (NEB, 5% SDS, 0.4 M DTT) were added to 13.5 μl of sample. This mix was incubated for 10 min at 95°C. After the sample had cooled down on ice, 2 μl G5 Buffer (NEB), 1 μl Endo H (NEB) and 2 μl ultrapure water were added and this mix was incubated for 60 min at 37°C. For the peptide: *N*-glycosidase F (PNGase F) digestion 1.5 μl of denaturing buffer were added to 13.5 μl of sample. This mix was incubated for 10 min at 95°C. After the sample had cooled down on ice, 2 μl G7 Buffer (NEB), 1 μl PNGase F (NEB), and 2 μl NP-40 were added and this mix was incubated for 60 min at 37°C.

### *N*- and *O*-Glycan Analysis

To analyze the sIgA1 *N*- and *O*-glycans, purified protein (1–5 μg) was separated by SDS-PAGE under reducing conditions, and polypeptides were detected by Coomassie Brilliant Blue staining. The corresponding band was excised from the gel, followed by *S*-alkylation with iodoacetamide and digestion with sequencing grade modified trypsin (Promega) or a combination of trypsin and endoproteinase Glu-C (Roche). The peptide mixture was analyzed using a Dionex Ultimate 3000 system directly linked to a QTOF instrument (maXis 4G ETD, Bruker) equipped with the standard ESI source in the positive ion, DDA mode (=switching to MS/MS mode for eluting peaks). MS-scans were recorded (range: 150–2200 m/z, spectra rate: 1 Hz) and the six highest peaks were selected for fragmentation. Instrument calibration was performed using ESI calibration mixture (Agilent). For separation of the peptides a Thermo BioBasic C18 separation column (5 μm particle size, 150 × 0.360 mm) was used. A gradient from 97% solvent A and 3% solvent B (Solvent A: 65 mM ammonium formiate buffer, B: 100% acetonitrile) to 32% B in 45 min was applied, followed by a 15 min gradient from 32% B to 75% B, at a flow rate of 6 μL/min.

The analysis files were converted to XML files using Data Analysis 4.0 (Bruker) and used to perform MS/MS ion searches with MASCOT (embedded in ProteinScape 3.0, Bruker) using the manually annotated and reviewed Swiss-Prot database. Peptide MS/MS data were evaluated against the target sequence using X! Tandem (www.thegpm.org/tandem/) with the following settings: reversed sequences no; check parent ions for charges 1, 2, and 3 yes; models found with peptide log e lower -1 and proteins log e lower -1; residue modifications: oxidation M, W and deamidation N, Q; isotope error was considered; fragment type was set to monoisotopic; refinement was used with standard parameters; fragment mass error of 0.1 Da and ±7ppm parent mass error; fragment types b and y ions; maximum parent ion charge of 4; missed cleavage sites allowed was set to 2; semi-cleavage yes.

### Jacalin Purification

Jacalin/Agarose (InvivoGen) was washed three times with PBS and centrifuged at 1500 × *g* for 4 min. SSL7/Agarose-purified IgA1 was diluted with PBS, added to the washed Jacalin/Agarose and incubated for 1.5 h at 4°C with slowly inverting. After incubation, the mix was centrifuged at 3220 × *g* for 10 min, the supernatant was removed and the Jacalin/Agarose was transferred to a spin column. The agarose was washed three times with 500 μl PBS and subsequent centrifugation at 1500 × *g* for 1 min. The bound protein was eluted by the addition of 50 μl elution buffer containing 0.1 M α-D-galactose in PBS and subjected to SDS-PAGE and immunoblotting.

## Results

### Transient Expression of Recombinant sIgA1 in *N. benthamiana* Wild-Type and Glyco-Engineered ΔXT/FT Plants

**FIGURE 2:**
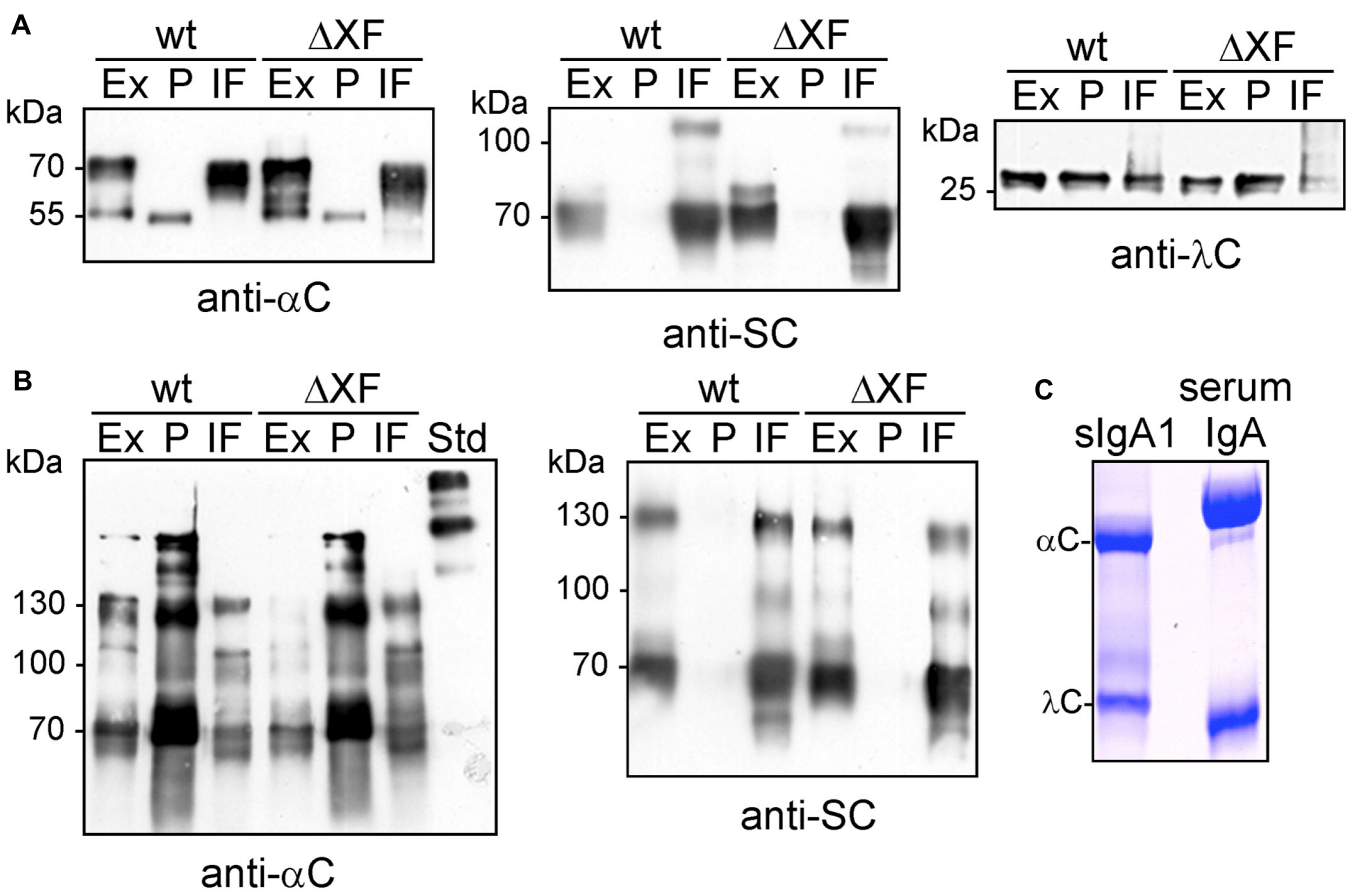
**Analysis of sIgA1 expression.** **(A)** SDS-PAGE and immunoblotting of crude protein extracts (Ex), SSL7-purified sIgA1 (P), and intercellular fluid (IF) from *N. benthamiana* wild-type (wt) or ΔXT/FT (ΔXF) infiltrated plants with either anti-alpha chain (anti-αC), anti-secretory component (anti-SC), or anti-lambda light chain (anti-λC) antibodies. **(B)** SDS-PAGE under non-reducing conditions followed by immunoblotting. **(C)** SDS-PAGE under reducing conditions and Coomassie staining of SSL-purified sIgA1 from plants. Human serum IgA was loaded for comparison.

Recombinant sIgA1 against rotavirus was transiently expressed via agro-infiltration in *N. benthamiana* wild-type and the glyco-engineered ΔXT/FT plants using the previously described GoldenBraid multigene expression system (Juarez et al., 2013). To obtain efficient co-expression of all proteins the four transcriptional units encoding the IgA1 alpha chain (αC), the lambda light chain (λC), the human SC, and the human JC were expressed from a single vector (**Figure 1B**). Extracts from leaves were taken three days after infiltration and subjected to SDS-PAGE and immunoblotting. Bands corresponding to the expected size of the alpha chain (∼55 kDa), light chain (∼23 kDa), and the SC (∼68 kDa) were found (**Figure 2A**). By contrast, the JC could not be detected on immunoblots (data not shown). The used anti-alpha chain antisera reacted not only with the alpha chain, but also with the SC. SDS-PAGE and immunoblotting under non-reducing conditions revealed the presence of presumably monomeric IgA1 variants (signals larger than 130 kDa and co-migrating with the lower bands of the standard) in the total protein extract. Additional bands at approximately ∼70 and 130 kDa were also detected with the antibody against the SC and very likely represent free monomeric and dimeric SC. Higher molecular weight complexes resembling dimeric or sIgA1 (compare with the top bands of the standard) were hardly detectable in all tested protein fractions (**Figure 2B**). Importantly, these observations were similar in wild-type as well as in glyco-engineered ΔXT/FT plants.

To further characterize the IgA variants, we purified them from leaves using binding to SSL7-agarose and investigated the presence of different IgA chains in the IF. The SSL7-purified protein consisted mainly of the alpha chain and the light chain (**Figure 2C**), no additional band corresponding to the J chain (∼17 kDa) was detected. The IF displayed the SC as predominant sIgA1-derived protein band (**Figure 2A**). In addition to the band corresponding to the SC, a faint unidentified additional band (slightly larger than 100 kDa) was also detected with anti-SC antibody. Interestingly, the alpha chain was not found in the IF suggesting that the monomeric IgA1 is not secreted to the apoplast. Together these findings indicate that the expressed sIgA1 is not efficiently assembled under the used conditions and that unassembled SC and some unincorporated lambda light chain are secreted to the apoplast.

### Characterization of *N*-Glycosylation Status of the Alpha Chain and Secretory Component

**FIGURE 3:**
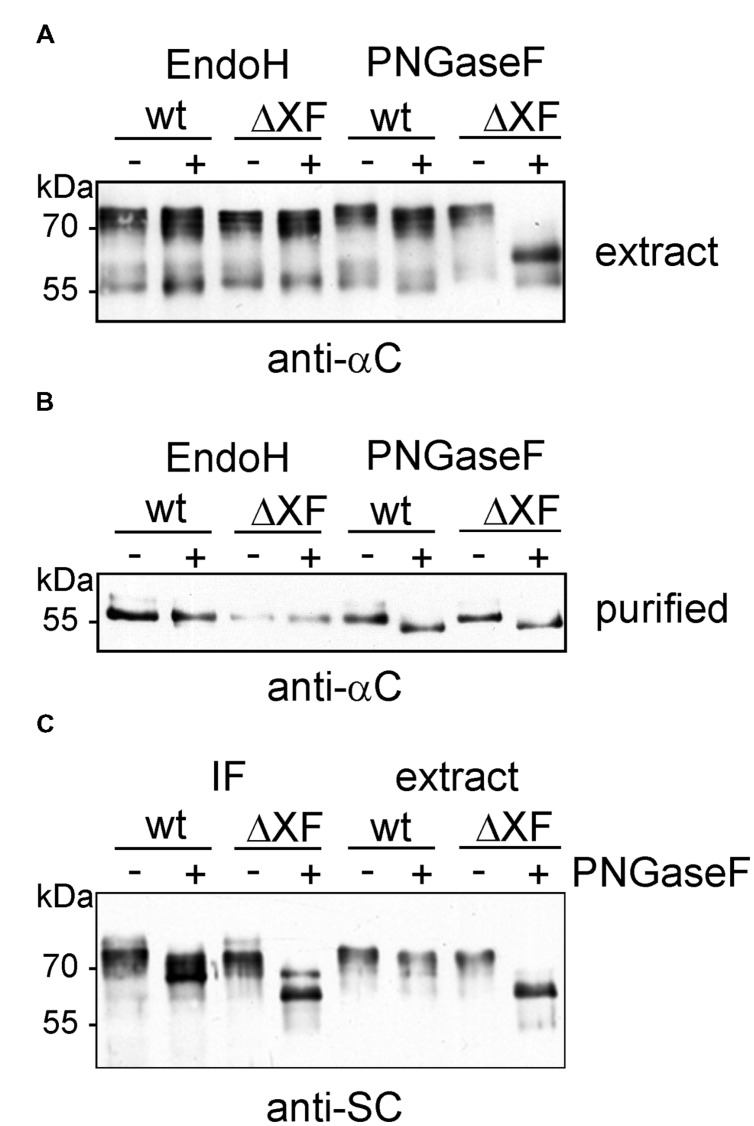
**Enzymatic deglycosylation of expressed sIgA1 chains.** **(A)** Crude protein extracts were digested with Endo H or PNGase F. Proteins were separated by SDS-PAGE and analyzed by immunoblotting with antibodies against the alpha chain (anti-αC). **(B)** SSL7-purified samples were Endo H and PNGase F digested, respectively, subjected to SDS-PAGE followed by immunoblotting with antibodies against the alpha chain. **(C)** IF and crude extracts were digested with PNGase F and analyzed by SDS-PAGE and immunoblotting with antibodies against the secretory component (anti-SC).

Next, we examined the glycosylation status of the alpha chain and the SC by endoglycosidase digestions and subsequent SDS-PAGE and immunoblotting. Extracts from infiltrated wild-type and ΔXT/FT leaves were digested with Endo H and PNGase F to distinguish between oligomannosidic (Endo H and PNGase F sensitive), core fucose-free complex (Endo H resistant, PNGase F sensitive), and core fucose-containing *N*-glycans (insensitive to both enzymes). While Endo H digestion of the alpha chain did not result in any mobility shift, a small shift was observed in the PNGase F digested samples (**Figure 3A**). The shift was comparable in wild-type and ΔXT/FT extracts indicating the presence of core fucose-free complex *N*-glycans on the IgA1 alpha chain. This result was confirmed by digestion of the SSL7-purified protein samples (**Figure 3B**). IF and leaf extracts were also analyzed for SC *N*-glycosylation. In wild-type, a small mobility shift was visible upon PNGase F digestion and immunoblotting in both the IF and the extract. By contrast, in ΔXT/FT the mobility shift was much larger indicating that the majority of *N*-glycans of the SC are core fucose-containing complex *N*-glycans (**Figure 3C**).

**FIGURE 4:**
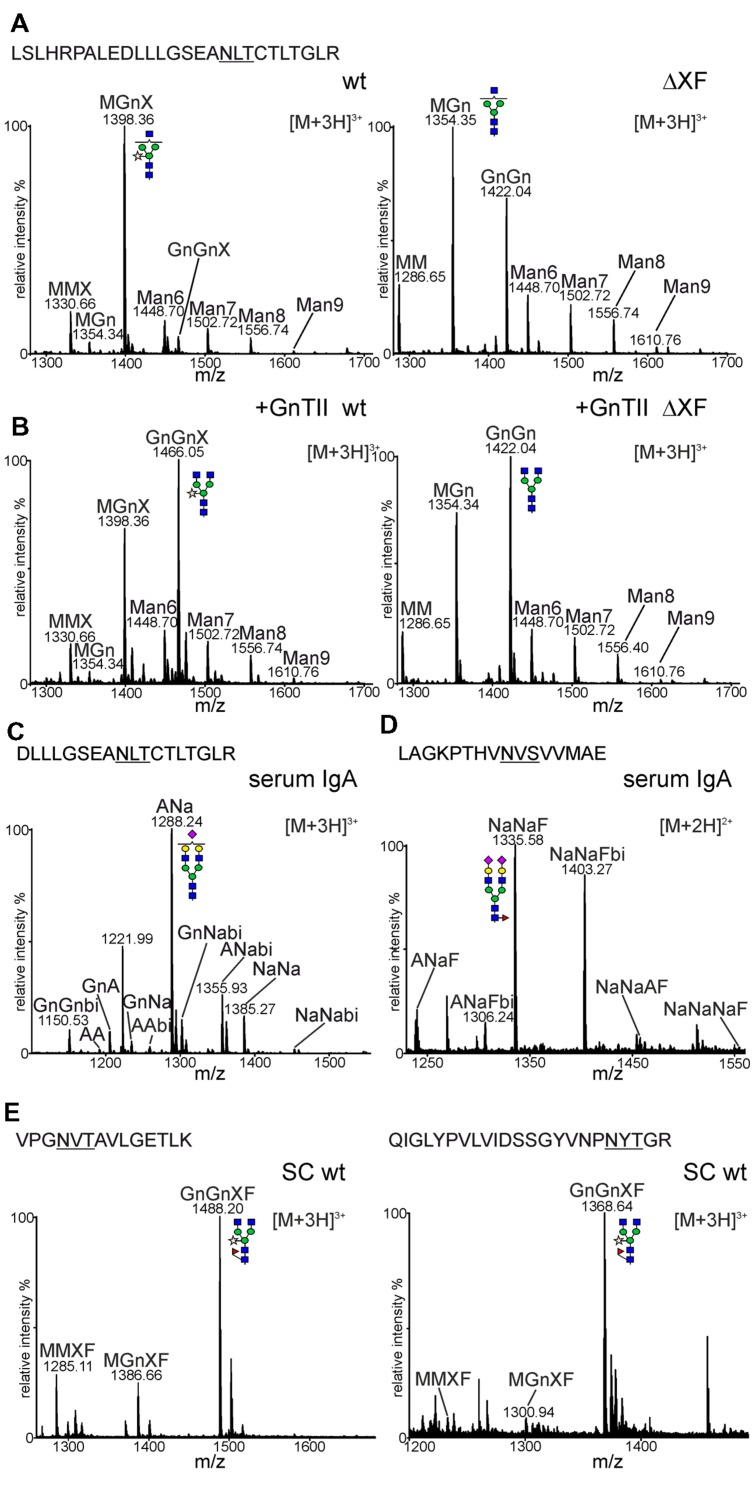
***N*-glycan analysis of sIgA1.** **(A)** Mass spectra of the tryptic glycopeptide from the CH2 domain of sIgA1 expressed in *N. benthamiana* wild-type (wt) or ΔXT/FT (ΔXF). The amino acid sequence of the identified peptide is highlighted. The *N*-glycosylation site is underlined. **(B)** sIgA1 was transiently co-expressed with human GnTII and analyzed as mentioned in **(A)**. **(C)** The corresponding Glu-C/trypsin digested glycopeptide from human serum IgA. **(D)** The spectrum of the tailpiece glycopeptide of the alpha chain from human serum IgA. **(E)** Spectra from two glycopeptides of the SC derived from the IF of wild-type plants. A detailed explanation of the used *N*-glycan abbreviations can be found at the ProGlycAn homepage (http://www.proglycan.com/index.php?page=pga_nomenclature). The graphical depictions of glycan-structures follow the style of the Consortium for Functional Glycomics (http://www.functionalglycomics.org/static/consortium/Nomenclature.shtml).

To determine the *N*-glycan composition more in detail, IgA1 was purified via SSL7-agarose and subjected to SDS-PAGE and Coomassie blue staining. The band corresponding to the alpha chain was excised, trypsin digested and peptides were analysed by LC-ESI-MS. The glycopeptide corresponding to the single *N*-glycosylation site in the CH2 domain was identified and found to harbor a single dominant peak (**Figure 4A**). The mass of this peak corresponds to a glycopeptide with a complex *N*-glycan furnished with a single terminal GlcNAc residue and a single pentose, presumably β1,2-linked xylose. Other peaks were reminiscent of a truncated glycan lacking terminal GlcNAc (MMX: Man_3_XylGlcNAc_2_) and different oligomannosidic (Man6 to Man9: Man_6_GlcNAc_2_ to Man_9_GlcNAc_2_) *N*-glycans. Fully processed complex *N*-glycans were only found in very low amounts (e.g., GnGnX: GlcNAc_2_Man_3_XylGlcNAc_2_; **Figure 4A**). Consistent with the PNGase F digestion, no fucose-containing peaks could be detected on the CH2 domain glycopeptide in *N. benthamiana* wild-type. The *N*-glycan profile from the ΔXT/FT-derived CH2 domain showed the incompletely processed MGn (GlcNAcMan_3_GlcNAc_2_) structure as the major peak and lower amounts of peaks corresponding to truncated (MM: Man_3_GlcNAc_2_), complex (GnGn: GlcNAc_2_Man_3_GlcNAc_2_) and oligomannosidic (Man6 to Man9) *N*-glycans. As expected, glycans with β1,2-xylose and core α1,3-fucose were not found in the glyco-engineered ΔXT/FT line.

The presence of large amounts of *N*-glycan structures with a single terminal GlcNAc residue in *N. benthamiana* suggests that the *N*-glycan in the CH2 domain is incompletely processed by the Golgi-resident GnTII or attached GlcNAc residues are cleaved off in post-Golgi compartments by β-hexosaminidases (Strasser et al., 2007; Castilho et al., 2014). To test the first possibility, human GnTII was cloned into a binary plant-expression vector (**Figure 1C**) and co-expressed with the sIgA1 multigene vector. As a result, peaks corresponding to complex *N*-glycans with two terminal GlcNAc residues were considerably increased in wild-type as well as in ΔXT/FT (**Figure 4B**). This result indicates that GnTII activity is a major limiting factor that leads to incompletely processed *N*-glycans on this *N*-glycosylation site of the IgA1 alpha chain. This limitation can at least in part be overcome by transient expression of the corresponding human glycosyltransferase.

In contrast to the plant-derived alpha chain *N*-glycans, the CH2 domain glycopeptide from a human serum standard displayed processing on both branches resulting in the formation of sialylated and galactosylated biantennary *N*-glycans (**Figure 4C**). The glycopeptide derived from the alpha chain tailpiece could also be identified in the human IgA standard. All identified peaks were sialylated and contained fucose (**Figure 4D**). Despite several attempts using different proteolytic digestions (trypsin or Glu-C plus trypsin), a peptide or glycopeptide containing the second *N*-glycosylation site in the alpha chain tailpiece could not be identified in our plant-derived samples. Consequently, the *N*-glycosylation status of this site remains unknown.

Since the SC could not be co-purified by SSL7-affinity purification we isolated IF from leaves of wild-type plants, extracted the corresponding band from SDS-PAGE and analyzed the trypsin digested sample for glycopeptides. In total four glycopeptides from the SC were identified, one of them harboring two glycosylation sites. All identified glycopeptides displayed a similar *N*-glycan profile (**Figure 4E** and data not shown) with a major peak corresponding to GnGnXF (GlcNAc_2_Man_3_XylFucGlcNAc_2_) and smaller amounts of incompletely processed (MGnXF: GlcNAcMan_3_XylFucGlcNAc_2_) and truncated *N*-glycans (MMXF: Man_3_XylFucGlcNAc_2_). All these *N*-glycans were processed in the Golgi and contained xylose and fucose residues.

### *O*-Glycan Analysis of the IgA1 Hinge Region

**FIGURE 5:**
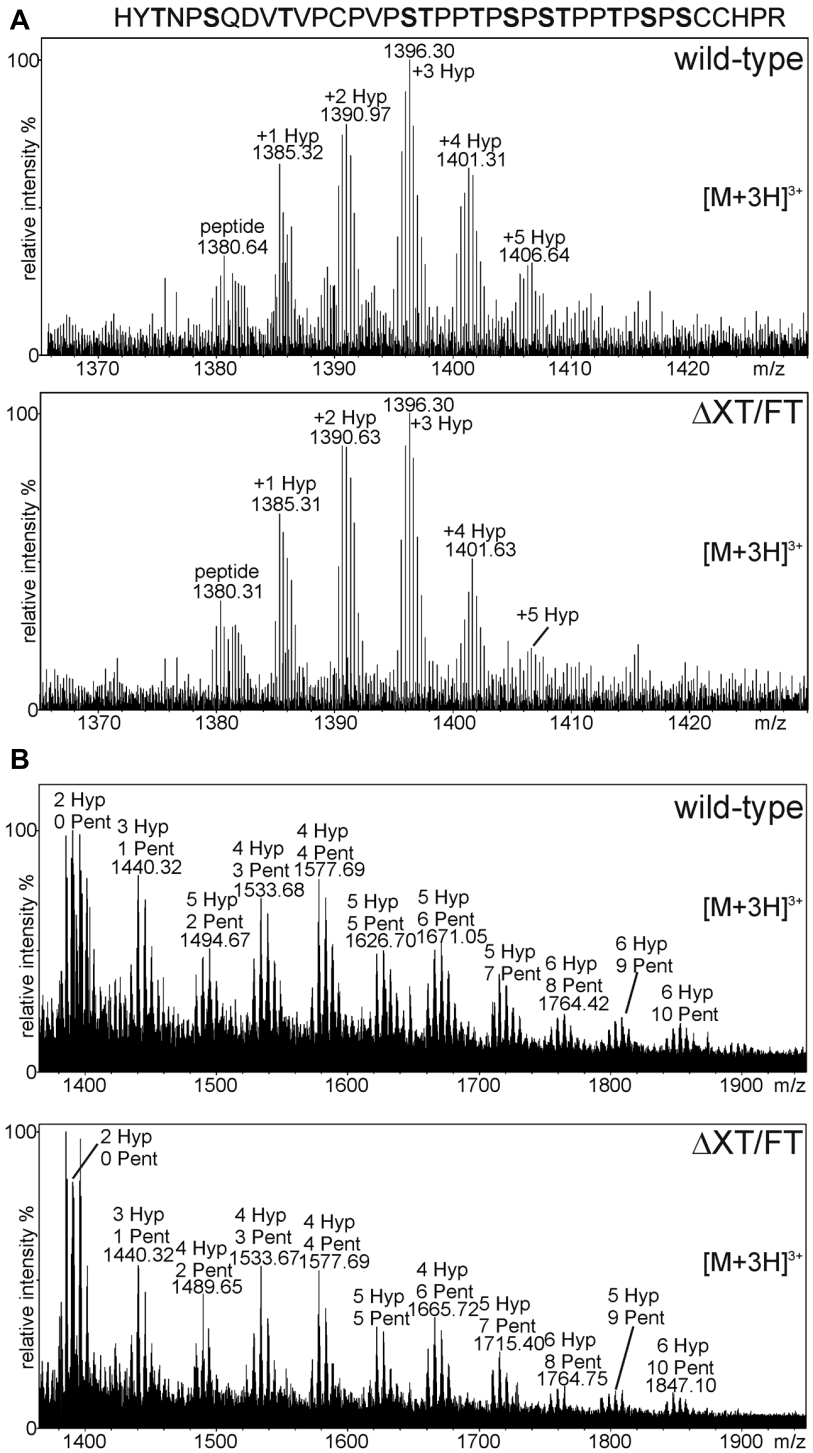
**MS analysis showing the presence of plant specific *O*-glycan modifications.** The analyzed peptide sequence with potential *O*-glycosylation sites (highlighted in bold) is shown. **(A)** The glycopeptide corresponding to the alpha chain hinge region contains different amounts of hydroxyproline (Hyp) residues. **(B)** Peaks corresponding to Hyp + pentoses (Pent) were detected in wild-type and ΔXT/FT plants.

Plant *O*-glycosylation differs significantly from mammals as plants do not have a functional mucin-type *O*-glycosylation pathway (Castilho et al., 2012; Yang et al., 2012). Plants, on the other hand, can convert proline residues adjacent to *O*-glycosylation sites into hydroxyproline (Hyp; Taylor et al., 2012). Serine residues next to specific Hyp-sequence motifs may be modified with single galactose and Hyp residues and can be extensively modified with arabinose chains or arabinogalactans (Seifert and Roberts, 2007; Basu et al., 2013; Saito et al., 2014). The presence of Hyp and arabinose chains in the hinge region has been described previously for maize seed-derived human IgA1 (Karnoup et al., 2005). To monitor the prolyl-hydroxylation and potential plant-specific *O*-glycosylation we analyzed the glycopeptide corresponding to the hinge region from IgA1 expressed in *N. benthamiana* wild-type and ΔXT/FT plants. For this purpose, transiently expressed IgA1 was purified from leaves using SSL7-agarose and tryptic peptides were subjected to mass spectrometric analysis. The peptide derived from the IgA1 alpha chain (HYTNPSQDVTVPCPVPSTPPTPSPSTPPTPSPSCCHPR) was analyzed for the presence of post-translational modifications. In **Figure 5A**, the spectra with peaks assigned to proline/Hyp conversions are shown. The observed heterogeneity in the MS-spectra indicates that proline residues in this region are partially converted into Hyp by plant prolyl-hydroxylases. A search for glycosylated variants of the peptide revealed the presence of modifications corresponding to the incorporation of pentose sugars (presumably arabinoses; **Figure 5B**). The modifications were comparable between wild-type and ΔXT/FT plants being in agreement with the hypothesis that modulation of the *N*-glycan processing pathway does not interfere with *O*-glycan modifications.

### Generation of Mucin-Type *O*-Glycans on Plant-Expressed Human IgA1

**FIGURE 6:**
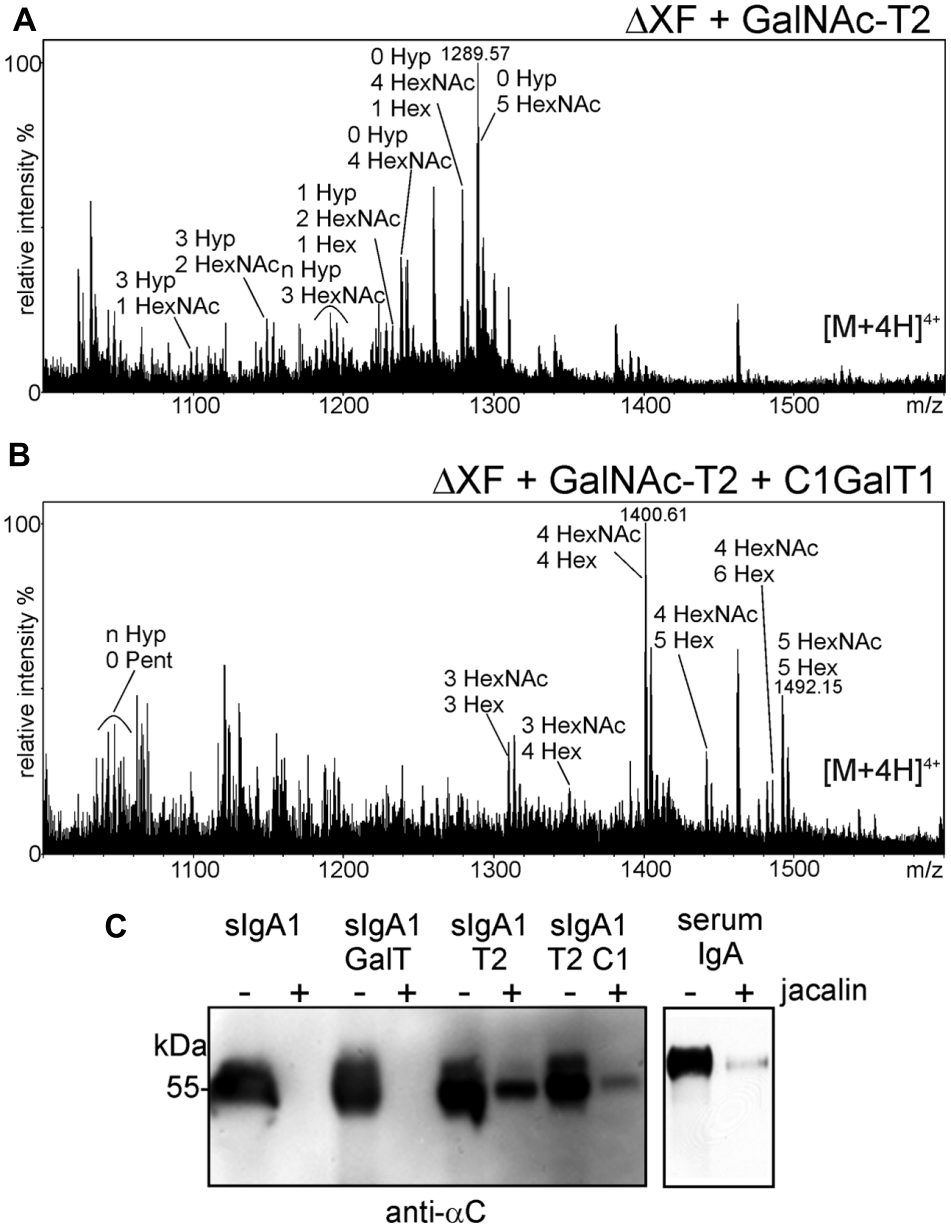
**Co-expression of GalNAc-T2 and C1GalT1 results in the modification of the hinge region peptide.** **(A)** Co-expression of GalNAc-T2 leads to the incorporation of HexNAc residues. **(B)** Co-expression of GalNAc-T2 and C1GalT1 results in the generation of core 1 structures: HexNAc + hexose (Hex). **(C)** Incubation with jacalin agarose (+) reveals binding of the sIgA1 alpha chain when modified *in planta* with mucin-type *O*-glycan biosynthesis enzymes. The amount of sIgA1 before binding to jacalin is also shown (-). Human serum IgA was used as a positive control and sIgA1 alone or co-expressed with the *N*-glycan modifying β1,4-galactosyltransferase (GalT) was used as negative control.

Three to six mucin-type *O*-glycans are commonly attached to the nine potential *O*-glycosylation sites in the hinge region of human IgA1. The major structures are mucin-type core 1 and sialylated core 1 *O*-glycans. In IgA nephropathy, a long term chronic kidney disease in humans, *O*-glycans are mostly galactose-deficient and recognized by anti-glycan antibodies leading to unwanted immune complex formation. For therapeutic applications it is therefore crucial to produce recombinant IgA variants with human-type sialylated core 1 *O*-glycans to avoid any adverse side effects and loss of functionality (Suzuki et al., 2015). To investigate whether the hinge region of human IgA1 can be furnished with defined mucin-type *O*-glycans when expressed in *N. benthamiana*, we performed *O*-glycan engineering. To this end, sIgA1 was co-expressed with different enzymes for initiation and elongation of mucin-type *O*-glycans (**Figure 1D**). For initiation of mucin-type *O*-glycosylation we chose to express human GalNAc-T2 which we have previously used for *in planta O*-glycan biosynthesis on the single *O*-glycosylation site of EPO-Fc (Castilho et al., 2012). As can be seen in **Figure 6A**, co-expression of sIgA1 with this single human enzyme (without any additional mammalian proteins like a UDP-GalNAc transporter) resulted in modification of the hinge region peptide with an additional HexNAc monosaccharide (Tn antigen-like structure). Co-expression of the *Drosophila melanogaster* β1,3-galactosyltransferase (C1GalT1) led to the incorporation of additional hexoses suggesting the successful formation of core 1 *O*-glycan structures (T antigen; **Figure 6B**).

The T antigen structure is recognized by the lectin jacalin which is commonly used for purification of human IgA1. Previously it was demonstrated that recombinant jacalin does not react with plant-produced sIgA1 which is normally devoid of any galactose or GalNAc residues (Fernandez-del-Carmen et al., 2013). Here, we tested whether a commercially available jacalin recognizes glyco-engineered sIgA1 purified from plants. Jacalin-agarose was incubated with different sIgA1 glycoforms and binding was tested by immunoblotting. Jacalin binding was observed for sIgA1 modified with GalNAc-T2 and for sIgA1 modified with GalNAc-T2 and C1GalT1. By contrast, no binding was observed for unmodified sIgA1 or for sIgA1 that was co-expressed with human β1,4-galactosyltransferase (Strasser et al., 2009) that acts predominately on *N*-glycans (**Figure 6C**).

**FIGURE 7:**
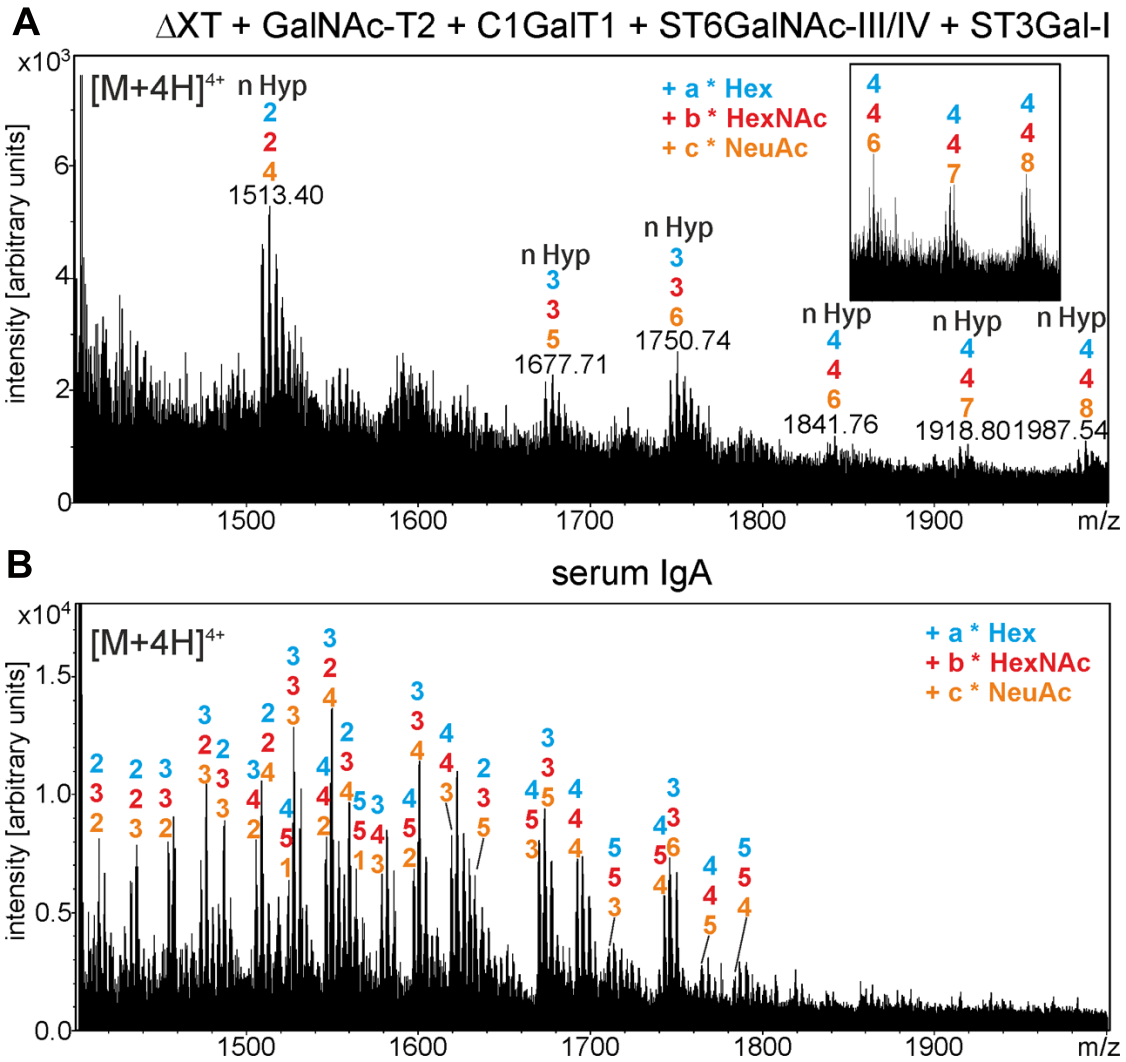
***In planta* generation of sialylated core 1 structures on the alpha chain hinge region.** **(A)** Mass-spectrum of trypsin-digested sIgA1 expressed in *N. benthamiana* ΔXT/FT (ΔXT) line is shown. sIgA1 was coexpressed with GalNAc-T2, C1GalT1, ST6GalNAc-III/IV, ST3Gal-I and the required proteins for CMP-sialic acid synthesis and Golgi-transport to generate disialyl core 1 structures. The inset shows peaks corresponding to modifications of four *O*-glycosylation sites with sialic acid (4–4–8: 4x hexose – 4x HexNAc – 8x *N*-acetylneuraminic acid). **(B)** For comparison, the *O*-glycan structures derived from human serum IgA are indicated.

Finally, to generate disialylated core 1 structures, the predominant *O*-glycan on serum-derived IgA1, we co-expressed sIgA1 with GalNAc-T2, C1GalT1, and the mammalian sialic acid biosynthesis pathway consisting of three enzymes (UDP-*N*-acetylglucosamine 2-epimerase/*N*-acetylmannosamine kinase; *N*-acetylneuraminic acid phosphate synthase and CMP-*N*-acetylneuraminic acid synthetase) for CMP-sialic acid formation and the CMP-sialic acid transporter for transport of the activated nucleotide sugar into the Golgi (Castilho et al., 2012). The MS-spectra showed distinct peaks corresponding to the generation of structures with HexNAc, hexoses, and *N*-acetylneuraminic acid (NeuAc; **Figure 7A**), which are similar to the structures found on human serum IgA1 (**Figure 7B**). In summary, these data show that different mucin-type *O*-glycans can be successfully generated on the hinge region of *N. benthamiana*-expressed sIgA1.

## Discussion

Glycosylation of the single IgG Fc-*N*-glycan has a huge impact on Fc-receptor binding leading to alterations in effector functions such as antibody dependent cellular cytotoxicity (ADCC). While the relevance of antibody glycosylation for effector functions has been realized some time ago (Lifely et al., 1995; Umaña et al., 1999), more recent *in vivo* and *in vitro* glyco-engineering approaches have resulted in a much deeper understanding of antibody glycan-structure-function relationships (Forthal et al., 2010; Ferrara et al., 2011; Ahmed et al., 2014; Lin et al., 2015; Subedi and Barb, 2015). As a consequence the Fc glycans are now categorized as critical quality attributes by industry (Reusch and Tejada, 2015). Despite this documented importance for IgG the role of glycosylation for other immunoglobulins is less well understood. Crucial for further developments and novel applications are suitable tools to manipulate and control the glycan composition on different immunoglobulins including IgMs and IgAs. Glyco-engineering has been very successfully applied to plants in the past (Strasser et al., 2014) and the great potential of *N. benthamiana* for production of therapeutic IgMs has recently been demonstrated (Loos et al., 2014). Here, we characterized *N*- and *O*-glycans from sIgA1 produced in wild-type and glyco-engineered ΔXT/FT plants and provided strategies toward the formation of defined *N*- as well as *O*-glycans that can be used in the future for extensive functional studies.

We initially aimed to produce a sIgA1 variant and co-expressed all four involved protein chains from a single expression construct (Juarez et al., 2013). Unexpectedly, we obtained mainly monomeric IgA1 variants indicating that the assembly to full sIgA1 variants was not efficient. One possible factor that influences the formation of dimeric and sIgA variants could be the limited expression of the J chain. In our immunoblot experiments, we were not able to monitor the expression of the J chain. Even the additional co-expression of the human J-chain did not result in a detectable J chain incorporation (data not shown). Alternatively, the fate of the alpha chain tailpiece could also affect the assembly of the dimeric or secretory form. We obtained good coverage of the human IgA1 alpha chain in the proteolytically digested peptide pools (data not shown), but were not able to detect the glycosylated or unglycosylated peptide corresponding to the C-terminal tailpiece. While we might have missed the (glyco)peptide during analysis it is also possible that the C-terminal end of the alpha chain is cleaved off in plants. Another recent study has detected difference in IgA alpha chain mobility by immunoblotting which was proposed to result from partial *N*-glycosylation of the tailpiece when transiently expressed in *N. benthamiana* (Westerhof et al., 2014). As previous studies in mammals have indicated an important role of the *N*-glycan in the tailpiece for J chain incorporation (Atkin et al., 1996; Sørensen et al., 2000), an effect of the altered C-terminal end on sIgA1 formation is plausible. Future studies will aim to address the nature of *N*-glycosylation in the tailpiece and its contribution for dimeric and sIgA1 formation in plants.

Analysis of the IF revealed that only the SC and light chain are present in considerable amounts, while most of the alpha chain and assembled IgA1 remains in the cells. A similar scenario of inefficient secretion of assembled IgA variants has been described for three IgA1 variants when transiently expressed in *N. benthamiana* wild-type (Westerhof et al., 2014). While the final subcellular location of the described IgA1 antibodies was not determined, another study reported the accumulation of sIgA mainly in the vacuoles of *N. benthamiana* leaves (Paul et al., 2014). However, in contrast to our findings, the sIgA in the later study showed predominately oligomannosidic *N*-glycans indicating a different subcellular trafficking route that bypasses the Golgi apparatus. We detected high amounts of truncated or incompletely processed complex *N*-glycans lacking core fucose on the *N*-glycosylation site in the CH2 domain of the alpha chain. In wild-type *N. benthamiana*, recombinant glycoproteins such as IgGs (Strasser et al., 2009), EPO-Fc (Castilho et al., 2012), α1-antitrypsin (Castilho et al., 2014), or IgM (Loos et al., 2014) that travel through the Golgi are very efficiently processed and frequently modified with both β1,2-xylose and core α1,3-fucose residues. Although not directly shown by site-specific glycopeptide analysis, the lack of core α1,3-fucosylation on IgA1 has also been observed based on PNGase F digestions and immunoblots by Westerhof et al. (2014). This uncommon lack of core α1,3-fucosylation is very likely caused by protein intrinsic features and less dependent on the expression host as the same glycopeptide from human serum IgA (**Figure 4C**) displays also reduced levels of core fucose. Moreover, recombinant IgA1 produced in murine myeloma or Chinese hamster ovary cells harbors also considerable amounts of complex *N*-glycans devoid of core fucose (Yoo et al., 2010). Interestingly, a supportive role of core α1,3-fucosylation on IgG Fc-glycan processing was recently discovered by analysis of the plant-produced cetuximab IgG1 antibody (Castilho et al., 2015).

Another difference to recombinant IgG expressed in *N. benthamiana* is the presence of incompletely processed or truncated complex *N*-glycans. These structures may derive either from inefficient GnTII activity during *N*-glycan processing in the Golgi or from post-Golgi action of plant β-hexosaminidases (Strasser et al., 2007). Co-expression of human GnTII converted a significant portion of the oligosaccharide into fully processed complex *N*-glycans indicating a limitation in endogenous GnTII activity. Whether β-hexosaminidases play an additional role in generation of *N*-glycan microheterogeneity like it has been shown for α1-antitrypsin (Castilho et al., 2014) remains to be shown in the future.

### *O*-Glycan Engineering: Challenges and Future Goals

The analysis of the IgA1 hinge region from wild-type or ΔXT/FT plants revealed the presence of plant-type Hyp formation and minor amounts of additional sugar residues. These posttranslational modifications have also been previously described for recombinant IgA1 expressed in maize seeds (Karnoup et al., 2005), on EPO-Fc (Castilho et al., 2012), on mucin-type glycopeptides expressed in *N. benthamiana* (Pinkhasov et al., 2011; Yang et al., 2012) and on moss-produced EPO (Parsons et al., 2013). All of these proteins have exposed proline residues next to *O*-glycosylation sites. The presence of these non-human modifications on therapeutic IgA1s may significantly affect the product quality and cause unwanted immune reactions. Therefore strategies are needed to avoid Hyp formation. The most promising approach is the elimination of the responsible prolyl-4-hydroxylase activity. Targeted disruption of a specific prolyl-4-hydroxylase in moss resulted in the removal of prolyl-hydroxylation on moss-produced recombinant EPO (Parsons et al., 2013). Given the success of the xylosyl- and fucosyltransferase knockdown in ΔXT/FT (Strasser et al., 2008) a similar strategy should also be feasible for elimination of unwanted plant-specific modifications related to *O*-glycans.

Compared to *N*-glycans, engineering of mucin-type *O*-glycosylation is highly challenging as a mammalian-like mucin-type *O*-glycan biosynthesis pathway is absent from plants (Strasser, 2013). The *de novo* synthesis of defined *O*-glycan structures in plants requires the coordinated expression of several different mammalian proteins in the secretory pathway of plants. However, the knowledge of factors that control mucin-type *O*-glycan biosynthesis in mammals is incomplete (Bennett et al., 2012). The initiation of mucin-type *O*-glycosylation, for example, is carried out by the large family of mammalian polypeptide GalNAc-transferases with 20 members in humans. Efficient transfer of GalNAc to multiple sites often requires the activity of different polypeptide GalNAc-transferase isoforms. Despite some recent progress, the acceptor substrate specificity of individual members from this family is still largely unclear (Steentoft et al., 2013; Kong et al., 2015). Here, we used the human GalNAc-T2, which is the key enzyme for IgA1 *O*-glycosylation initiation (Iwasaki et al., 2003). Our findings indicate that GalNAc can be efficiently transferred to Ser/Thr residues present in the IgA1 alpha chain hinge region when co-expressed with human GalNAc-T2 in plants. While further elongation with galactose and sialic acid led to human IgA1-like *O*-glycan structures, the incorporation of sialic acid was not very efficient. In the future, the biosynthesis of disialylated core 1 *O*-glycans can be further optimized by the use of multigene vectors for expression of all glycosyltransferases, by more precise subcellular targeting of the mammalian enzymes (Strasser et al., 2014) as well as by the use of transgenic *N. benthamiana* lines that stably express parts of the pathway (e.g., all the genes for CMP-sialic acid synthesis; Castilho et al., 2008). Together these advances will pave the way for detailed functional analysis of individual IgA glycoforms with the ultimate aim to generate recombinant glycoprotein therapeutics in *N. benthamiana* with reduced adverse side effects and maximized efficacy.

## Author Contributions

MD, MT, DM, and JK performed the research, MD, MT, DM, PJ, DO, FA, HS, and RS provided analytical reagents/tools and analyzed data, MD, MT, and RS designed the research, and RS wrote the paper.

## Conflict of Interest Statement

The authors declare that the research was conducted in the absence of any commercial or financial relationships that could be construed as a potential conflict of interest.

## Abbreviations

GALNAc-T2: polypetide-*N*-acetylgalactosaminyltransferase 2
